# Experimental data for creep and dynamic mechanical properties of polycarbonate and polycarbonate/acrylonitrile-butadiene-styrene

**DOI:** 10.1016/j.dib.2022.108264

**Published:** 2022-05-11

**Authors:** Quanyi Mu

**Affiliations:** a Ningxia Key Laboratory of Intelligent Sensing for Desert Information, School of Physics and Electronic-Electrical Engineering, Ningxia University, Yinchuan 750021, People's Republic of China; b State Key Lab for Strength and Vibration of Mechanical Structures, School of Aerospace Science, Xi'an Jiaotong University, Xi'an 710049, People's Republic of China

**Keywords:** PC, PC/ABS, Long-term creep behavior, Viscoelastic, DMA, Superposition principle

## Abstract

The data available in this article presents the tensile creep behaviors and dynamic mechanical properties of polycarbonate (PC) and the alloy of polycarbonate/acrylonitrile-butadiene-styrene (PC/ABS). Optical method in conjunction with a universal testing machine was used to get the creep deformation, and finally obtained the creep strain-time curves at different stress levels. The tensile creep stress levels for PC were 52 MPa, 50 MPa, 49 MPa, 48 MPa, 47 MPa, 41 MPa, and 47 MPa, 45 MPa, 43 MPa, 41 MPa, 39 MPa, 30 MPa for PC/ABS. Furthermore, the microstructural images of the creep fracture surface of PC and PC/ABS at different stress levels were obtained by using scanning electron microscope (SEM). Finally, the dynamic mechanical raw data of PC and PC/ABS were tested using dynamic mechanical analysis (DMA), and the dynamic mechanical properties of PC/ABS at three frequencies (0.1 Hz, 1 Hz and 10 Hz) were presented. The creep data can be reused to predict the long-term creep behavior of PC and PC/ABS, either by modeling predictions or by using the superposition principle to construct a long-term creep master curve. The SEM images and dynamic mechanical data can facilitate the investigating of the viscoelastic mechanism of PC and PC/ABS. These data can also be reused for comparison with PC and PC/ABS manufactured using other methods, such as 3D printed or recycled.

## Specifications Table

**Table:** 

Subject	Materials Science
Specific subject area	Materials Mechanics, Material Characterization, Polymers and Plastics, Materials Science Engineering
Type of data	Figure Image Microsoft Excel worksheet (XLSX) file
How the data were acquired	The raw stress–strain data (Fig. 1) were obtained using the electromechanical testing frame (GMT-4104-G, Suns, China) at room temperature. The design of the specimens for tension test and creep test was according to ASTM-D-638-14. The creep strain-time curves (Figs. 2 and 3) were obtained by using optical method in conjunction with a universal testing machine (GMT-4104-G, Suns, China) [1]. The acquired images were analyzed using ImageJ software to calculate the creep strain. The microstructural images of the creep fracture surface (Figs. 4 and 5) were obtained by using a scanning electron microscope (SEM, Quantum 200, FEI, USA). The images were taken with an accelerating voltage of 20 kV. Dynamic mechanical data (Figs. 6 and 7) were obtained by using dynamic mechanical analysis (DMA, STDA816e, Mettler Toledo, Switzerland) in the single cantilever mode at a frequency of 0.1Hz, 1Hz, 10 Hz and an amplitude of 20 µm. The samples were heated from 30 °C to above 150 °C with a rising step of 2 °C/min.
Data format	Raw Analyzed
Description of data collection	Data was collected through a series of experimental investigations.
Data source location	Institution: Xi'an Jiaotong University City: Xi'an Country: People's Republic of China
Data accessibility	Repository name: Mendeley Data Data identification number: DOI: 10.17632/k2m7mjb8mc.1 Direct URL to data: https://data.mendeley.com/datasets/k2m7mjb8mc/1

## Value of the Data

• Measuring creep data is a bit time-consuming. These data provide an in-sight into the creep behavior of PC and PC/ABS under different stress levels. After creep tests, the microstructural images of the creep fracture surface were obtained by using a SEM. The SEM images and dynamic mechanical data can facilitate the studying of the viscoelastic mechanism of PC and PC/ABS.
• The information presented and appended with this article will be beneficial to researcher, who may include mechanics modeling creep behavior, as well as material scientists studying material modification of PC and PC/ABS and their additive manufacturing.
• The creep data can be reused to modeling and predicting long-term creep behavior of PC and PC/ABS [2]. The creep data and dynamic mechanical data can be reused to construct long-term master curves by using superposition principle [3,4].
• These data, especially the SEM images of the fracture surface, can be reused for comparison with PC and PC/ABS manufactured using other methods, such as those obtained from 3D printing or recycling [5,6].

## 1 Data Description

The collected data in this article are the stress–strain data, the tensile creep data and the dynamic mechanical data of PC and PC/ABS. The collected data includes 7 figures and raw data (organized in 6 files in a public repository called Mendeley Data).

**Fig. 1:**
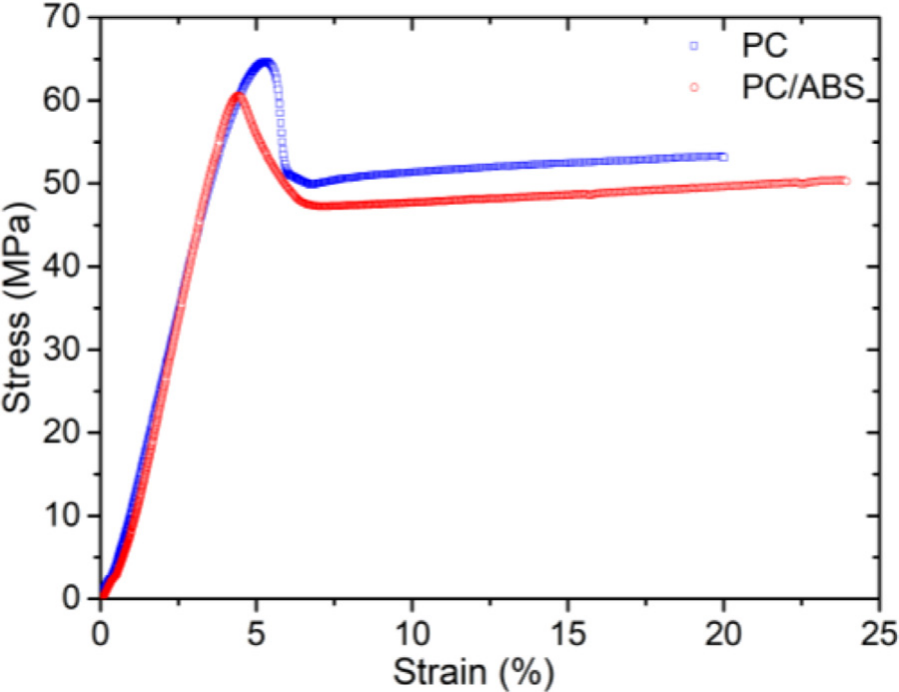
Stress–strain curves of the PC and PC/ABS.

We tested the stress–strain curves of the PC and PC/ABS (Fig. 1), and obtained the yield stress of them. The file name of the raw data corresponding to Fig. 1 is “Tension data_PC & PC-ABS.xlsx” in https://data.mendeley.com/datasets/k2m7mjb8mc/1.

**Fig. 2:**
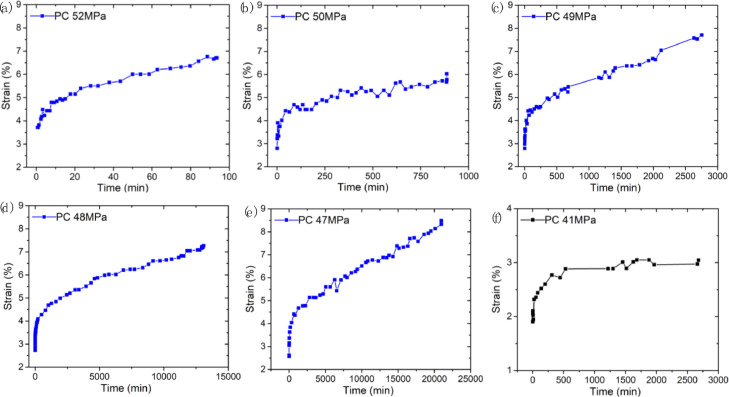
Tensile creep strain-time curves of PC at different stress levels: (a) 52 MPa, (b) 50 MPa, (c) 49 MPa, (d) 48 MPa, (e) 47 MPa, (f) 41 MPa (manually stopped the creep experiment at this low stress level).

**Fig. 3:**
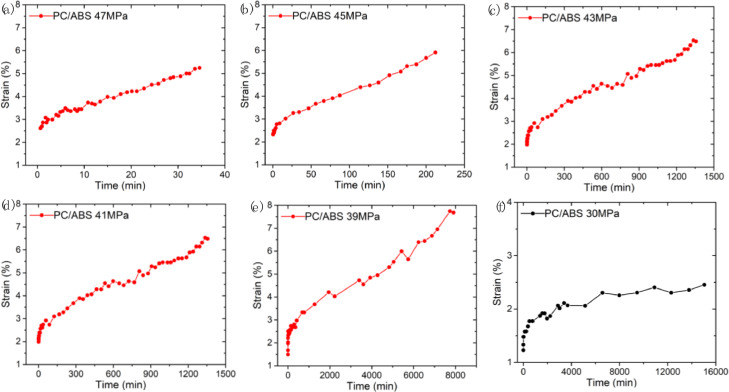
Tensile creep strain-time curves of PC/ABS at different stress levels: (a) 47 MPa, (b) 45 MPa, (c) 43 MPa, (d) 41 MPa, (e) 39 MPa, (f) 30 MPa (manually stopped the creep experiment at this low stress level).

For the creep test, a loading stress less than 0.8 times the yield stress was chosen, and reduced the loading stress by 1 MPa or 2 MPa each time according to the fracture time. Fig. 2. reports the tensile creep strain-time curves of PC at six stress levels: 52 MPa, 50 MPa, 49 MPa, 48 MPa, 47 MPa and 41 MPa, it should be noted that we manually stopped the creep experiment at the stress level of 41 MPa due to the long fracture time. Fig. 3. reports the tensile creep strain-time curves of PC/ABS at six stress levels: 47 MPa, 45 MPa, 43 MPa, 41 MPa, 39 MPa, and 30 MPa, we also stopped the creep experiment of PC/ABS at 30 MPa stress level due to the long fracture time. Data related to Figs. 2 and 3 are available in “Creep data_PC.xlsx” and “Creep data_PC-ABS.xlsx”, respectively, in https://data.mendeley.com/datasets/k2m7mjb8mc/1.

**Fig. 4:**
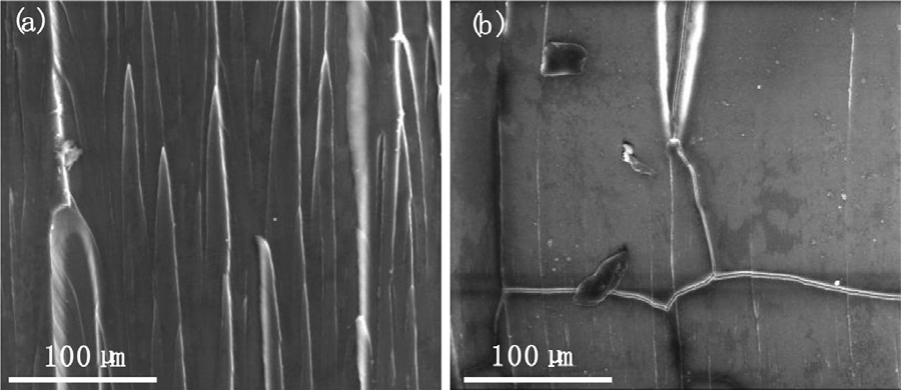
SEM images of the tensile creep fracture surfaces of PC (a) at a relatively low stress level of 47 MPa, (b) at a relatively high stress level of 52 MPa.

**Fig. 5:**
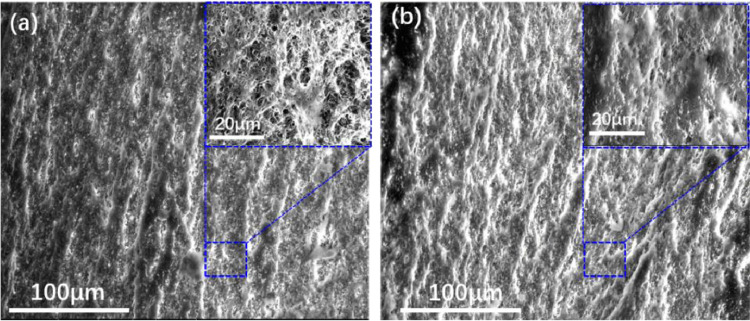
SEM images of the tensile creep fracture surfaces of PC/ABS (a) at a relatively low stress level of 39 MPa, (b) at a relatively high stress level of 47 MPa.

After the creep test, we performed microstructural analysis of the tensile creep fracture surfaces at different stress levels using SEM. Fig. 4. shows the SEM images of the tensile creep fracture surfaces of PC at 47 MPa and 52 MPa. Fig. 5. shows the SEM images of the tensile creep fracture surfaces of PC/ABS at 39 MPa and 47 MPa. See “SEM Image.zip” in https://data.mendeley.com/datasets/k2m7mjb8mc/1.

**Fig. 6:**
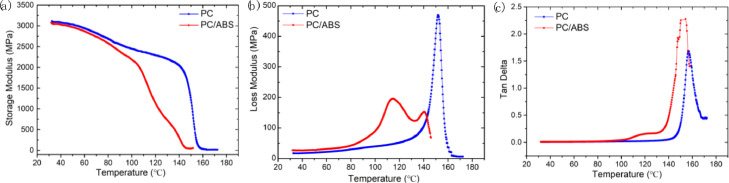
Dynamic mechanical properties of PC and PC/ABS: (a) Storage modulus, (b) Loss modulus, (c)Tan delta.

**Fig. 7:**
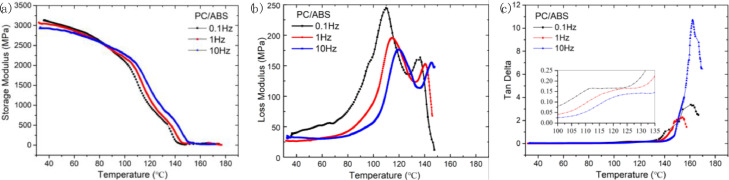
Variation of storage modulus, loss modulus and tan delta with temperature for PC/ABS at different frequencies: (a) Storage modulus, (b) Loss modulus, (c)Tan delta.

In addition, to further investigate the viscoelastic properties of PC and PC/ABS, we tested the dynamic mechanical properties of these two materials using DMA. Fig. 6. compared the storage modulus, the loss modulus and the tan delta curves of PC and PC/ABS. Fig. 7. shows the variation of the storage modulus and loss modulus, tan delta curves with temperature at different frequencies of PC/ABS. Raw dynamic mechanical properties data used in Figs. 6 and 7 are available in “DMA data_PC & PC-ABS.xlsx” and “DMA data_ PC-ABS_different frequence.xlsx”, respectively, in https://data.mendeley.com/datasets/k2m7mjb8mc/1.

## 2 Experimental Design, Materials and Methods

### 2.1 Materials and specimens

Neat PC and PC/ABS were used in this work. The average molecular weight of PC was 30,000 g/mol. PC/ABS has a blend ratio of 70% PC and 30% ABS in weight. Dog bone specimens with a gage length of 15 mm were machined and polished for the tensile tests and creep tests. Rectangular shape specimen with a size of 50 × 8 × 2.5 mm^3^ was used for the DMA tests.

### 2.2 Tensile creep experiment

Tensile tests and dead-load tensile creep tests were conducted using the electromechanical testing frame (GMT-4104-G, Suns, China) at room temperature. Tensile tests were performed with a crosshead speed of 2 mm/min. For the tensile creep experiments, the initial load was applied to the specimens at a speed of 3000 N/min, then the load was kept constant until the end of the experiment. The stress was defined as the tensile load divided by the initial cross-section area of the specimens. All tensile creep tests were performed at room temperature (∼22 °C)

### 2.3 Displace measurement

**Fig. 8:**
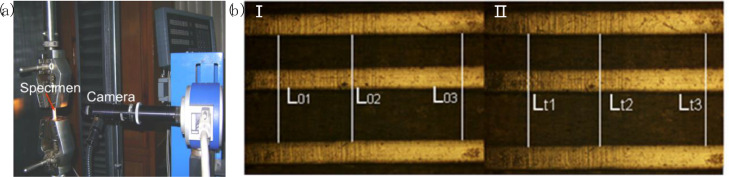
The measurement method for creep strain: (a) Pictures showing the deformation measurement system, (b) The photos of the surface of the specimen (Ⅰ) undeformed (Ⅱ) deformed.

To measure the creep deformation, several lines were marked on the surface of the specimen with a pencil before loading. As shown in Fig. 8a, a camera was used to take photos of the surface of the specimen, so that the deformation of the specimen during creep experiment can be recorded. A photo of the specimen surface was taken by the camera after clamped the specimen at the beginning of the experiment, and successive photos were taken during the experiment with the same magnification factor at certain time intervals. Fig. 8b shows the photos of the surface of the specimen and the deformation measurement method. Three pairs of feature points were chosen on the surface of the specimen, and the variation of the distance between each pair of the feature points during the creep experiment was measured by using the open source image processing software ImageJ. Creep strain was calculated with Eq. (1). The average value of the three calculated strains was taken as the strain at each moment.

(1) $$\varepsilon \left(t\right)=(L_{t}-L_{0})/L_{0}$$

Where *L_0_* is the initial length between each pairs of feature points that was measured on the undeformed surface of the specimen, *L_t_* is the corresponding lengths measured on the subsequently deformed surface of the specimen.

### 2.4 Dynamic mechanical test

Dynamic mechanical analysis (DMA) tester (STDA816^e^, Mettler Toledo, Switzerland) was used to characterize the dynamic mechanical properties of PC and PC/ABS in the single cantilever mode at a frequency of 1 Hz (0.1 Hz, 10 Hz) and an amplitude of 20 μm. The samples are heated from 30 °C to above 150 °C with a rising step of 2 °C/min under N_2_ atmosphere.

### 2.5 Other characterization

To investigate the microstructure of tensile creep fracture surfaces of PC and PC/ABS at different stress levels, scanning electron microscopic (SEM) images were acquired on a scanning electron microscope (Quantum 200, FEI, USA) with an accelerating voltage of 20 kV.

## Ethics Statements

No ethical issues are associated with this work.

## CRediT Author Statement

**Quanyi Mu**: Conceptualization, Investigation, Data curation, Writing- Reviewing and Editing.

## Declaration of Competing Interest

The author declares that no known competing financial interests or personal relationships that could have appeared to influence the work reported in this paper.

## Data Availability

Data for: Experimental data for creep and dynamic mechanical properties of polycarbonate and polycarbonate/acrylonitrile-butadiene-styrene (Original data) (Mendeley Data).
